# A Panel of CircRNAs in the Serum Serves as Biomarkers for *Mycobacterium tuberculosis* Infection

**DOI:** 10.3389/fmicb.2020.01215

**Published:** 2020-06-09

**Authors:** Hengjun Liu, Geng Lu, Weixiang Wang, Xinrui Jiang, Shuangshuang Gu, Jin Wang, Xin Yan, Fei He, Jun Wang

**Affiliations:** ^1^Department of Emergency, Nanjing Drum Tower Hospital, The Affiliated Hospital of Nanjing University Medical School, Nanjing, China; ^2^Department of Respiratory Medicine, Nanjing Drum Tower Hospital, The Affiliated Hospital of Nanjing University Medical School, Nanjing, China; ^3^School of Life Sciences, Nanjing University, Nanjing, China

**Keywords:** biomarker, circRNA, serum, tuberculosis, infection

## Abstract

Tuberculosis (TB), one of the ancient and deadliest diseases, is a chronic immune disorder caused by *Mycobacterium tuberculosis* (Mtb) infection. Due to the lack of ideal diagnostic and therapeutic markers, TB is still posing a major health, social, and economic burden worldwide. Circular RNA (circRNA), a newly discovered endogenous RNA, is abundant and stable in the cytoplasm and has tissue specificity. More and more studies suggested circRNA is involved in a variety of human pathological and physiological processes. Recently, several studies have confirmed circRNAs not only existed in the serum but also could serve as ideal biomarkers for detecting diseases since the circRNAs have continuous, stable, and covalently closed circular structures and are not easily degraded by nucleases. In this study, we screened the circRNA expression profiles in active TB serum samples and healthy volunteers serum samples by circRNA microarrays. Then, we performed qRT-PCR to verified the dysregulated circRNAs and ROC curve analysis to evaluate the value of circRNAs for TB diagnosis. The results showed circRNA_051239, circRNA_029965, and circRNA_404022 could serve as biomarkers for TB diagnosis.

## Introduction

Tuberculosis (TB) is the ninth leading cause of death worldwide with 1.5 million deaths and 10 million new cases worldwide in 2018 (Reinhart et al., 2012). Despite advances in the effective treatment of TB in recent years, the number of annual deaths and infections remains almost unchanged (Reinhart et al., 2012). The prevention, diagnosis and treatment of tuberculosis are mostly important for TB control. Since there are millions of infected people around the world, timely and effective isolation patients and treatment is the key factors to reduce the transmission of TB. This is based on the effectively simple and cheap early diagnosis of TB. Currently, the commonly used diagnosis technologies for TB are imaging inspection, bacteriological inspection, molecular biological detection, and immunological experimental inspection. However, all of these methods have some unavoidable limitation. For example, typical imaging inspection (such as X-ray) is useful for the diagnosis of TB, but the specificity is too low to distinguish the TB infection from the other lung disease, such as cancer, pneumonia (Bhalla et al., 2015). The bacteriological inspection is still the gold standard for TB diagnosis, but it requires 4–8 weeks for the growth of *M. tuberculosis*. Therefore, identifying novel appropriate biomarkers for early diagnosis of TB is an urgent.

Circular RNAs (circRNAs) are a novel class of RNAs that participate in almost all the physiological and pathological processes by acting as competing endogenous (ceRNAs) RNAs to block the functions of their target microRNAs (miRNAs) by binding target miRNAs to relieve the suppression of miRNAs for their target gene (Thomson and Dinger, 2016). CircRNAs are highly stable and resistant to debranching and exonuclease-mediated degradation since they could form a circular structure closed by covalent bonds. Recently, several studies have provided circRNAs could not only exist in the body fluids, such as plasma/serum, interstitial fluid, and saliva, but also could serve as molecular biomarkers in the diagnosis and prognosis of many diseases (Zhang et al., 2018). For example, Sonnenschein et al. (2019) found circDNAJC6, circTMEM56, and circMBOAT2 in the serum could distinguish between healthy and hypertrophic cardiomyopathy patients. Ye et al. (2019) identified hsa_circ_0082182, hsa_circ_0000370 were significantly upregulated in colorectal cancer plasma, and the hsa_circ_0035445 was down-regulated.

In this study, we firstly investigated the circRNA expression profiles in serum samples from active TB patients and healthy controls by circRNA microarrays. Subsequently, the dysregulated circRNAs was verified by qRT-PCR, and three circRNAs (circRNA_051239, circRNA_029965, and circRNA_404022) showed significantly increases in the serum from the active TB patients, compared to the healthy controls. The ROC curve analysis showed these three circRNAs could serve as biomarkers for TB diagnosis. More interestingly, we found circRNA_051239 was significantly upregulated in serum derived from drug-resistant TB patients compared to drug-susceptible patients.

## Materials and Methods

### Patients and Healthy Controls

**FIGURE 1:**
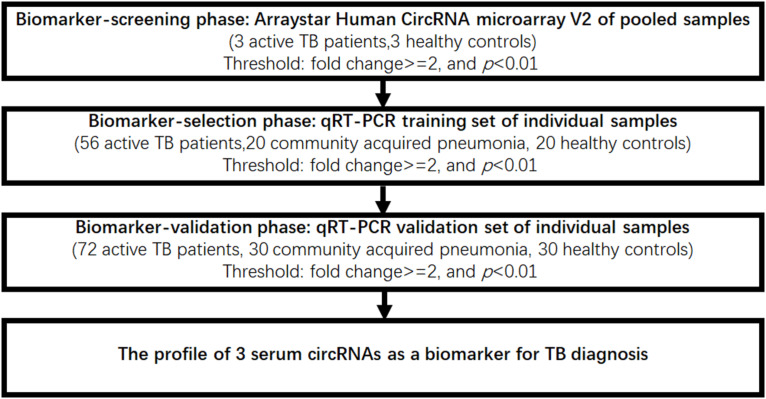
Flow chart of the experimental design.

Patients (*n* = 131) with active pulmonary TB, patients (*n* = 50) with community acquired pneumonia (CAP), and healthy controls (*n* = 53) were collected from the Nanjing Drum Tower Hospital. Serum samples were collected at the patients’ first admission to the hospital. All the TB patients were clinically diagnosed as active pulmonary TB by positive AFB smear staining or sputum culture. For CAP patients, they shared similar symptoms similar to TB infection (such as fever and chills, loss of appetite, shortness of breath), but didn’t have pulmonary TB by sputum smear or/and TB culture. The age- and sex-matched healthy controls, who had no clinical symptoms and normal physical examination, and sputum smear or/and TB culture for pulmonary TB were negative, were collected from the physical examination center of Nanjing Drum Tower Hospital. Patients and controls were matched based on age and gender. All the participants provided their written informed consent to participate in the study and the protocol was approved by the Institutional Research Board of the Nanjing Drum Tower Hospital. Protocols were designed and performed according to the principles of the Helsinki Declaration. Patient characteristics are summarized in Table 1. In order to identify serum circulating circRNAs which could serve as novel biomarkers for active TB diagnosis, a multiphase, case-control study was conducted (Figure 1).

**TABLE 1:** The demographic and clinical characteristics of active TB patients, community acquired pneumonia (CAP) patients, and healthy individuals in training and validation sets.

**Variable**	**TB (*n* = 128)**	**CAP (*n* = 50)**	**HC (*n* = 50)**
Age, years^a^	43.5 (19.84)	45.3 (18.25)	44.4 (18.43)
**Sex, n**			
Male	87	31	29
Female	41	19	21
**History of TB treatment**			
Yes	32		
No	96		

**^*a*^Age data are present as the mean (SD).**

### CircRNA Microarray

Total RNA was isolated from serum with TRIzol reagent (Invitrogen, Carlsbad, CA, United States) according to the manufacturer’s protocol. The concentration and quality of total RNA of each sample were determined with NanoDrop ND-1000 spectrophotometer (NanoDrop, Wilmington, DE, United States). CircRNA expression profiles of serum for three active pulmonary TB patients and three healthy controls were generated using the 074301 Arraystar Human CircRNA microarray V2. The data processing and quantile normalization were performed by R (Version 3.4.1) software.

### qRT-PCR

For qRT-PCR, cDNA was synthesized with SuperScript First-Strand Synthesis System (Invitrogen, Carlsbad, CA, United States) from RNA extracted from serum. Subsequently, qRT-PCR reaction was performed by an ABI 7500 real-time PCR System according to the manufacturer’s protocol. Briefly, PCR reactions were performed in a total volume of 10 μL, including 3 μL cDNA, 5 μL 2 × SYBR Green, 0.25 μL primer forward (10 μmol/L) and 0.25 μL primer reverse (10 μmol/L) at 95°C for 10 min, followed by 40 cycles of 95°C for 15 s and 60°C for 60 s. The synthetic *Caenorhabditis elegans* miRNA cel-miR-39 (5′-*UCACCGGGUGUAAAUCAGCUUG*-3′) (RiboBio, Guangzhou, China) was spiked into the serum as a normalization control (Kroh et al., 2010). The primers for circRNAs and cel-miR-39 were synthesized at Genscript (Nanjing, China). All reactions were analyzed in triplicate. Receiver operating characteristic (ROC) curve analysis was utilized to estimate the diagnostic value of serum circRNA.

### Expression and Statistical Analyses

SPSS 18.0 software was used for statistical analyses, and GraphPad Prism 6.0 (GraphPad Software, San Diego, CA, United States) was used to generate graphs. To compare significant differences in serum circRNA expression, the Mann-Whitney *U* test was used. A *P*-value of < 0.05 was regarded as statistically significant.

## Results

### Dysregulated CircRNAs Expression Profile in TB Patients

**FIGURE 2:**
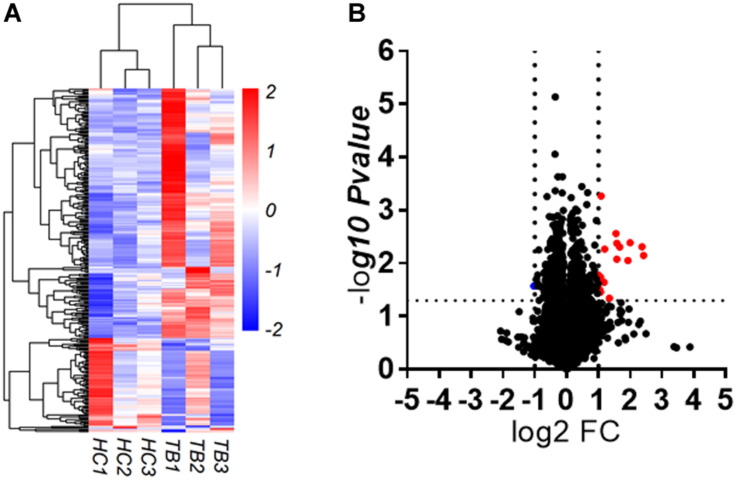
circRNAs expression in serum derived from active TB patients and healthy controls (HC). **(A)** Heatmap. **(B)** Volcano plot.

**FIGURE 3:**
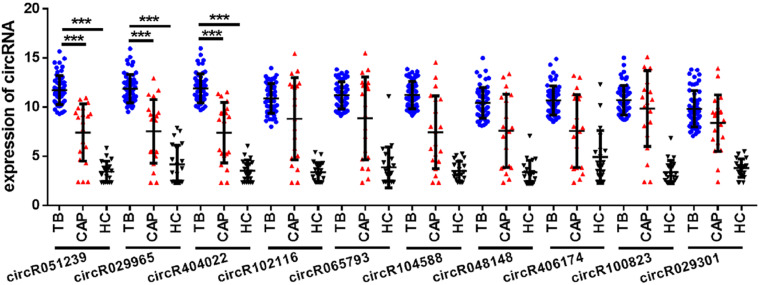
The expression of circRNAs in serum from active TB patients (*N* = 56), CAP patients (*N* = 20), and healthy controls (*N* = 20) by qRT-PCR. Each point represents the mean of triplicate samples. Each *P*-value was calculated with a nonparametric Mann-Whitney test. ***P < 0.001.

To explore whether serum circRNAs are potential biomarkers for TB infection, the landscape of expressed circular transcripts and their expression pattern in serum was investigated by performing the Agilent-069978 Arraystar Human CircRNA microarray V1 for six serum samples (three active TB patients and three healthy controls) in the biomarker-screening phase (Supplementary Table S1). As showed in Figures 2A,B, Hierarchical clustering and scatter plots indicated that the serum circRNA expression patterns had the potential possibility to distinguish TB patients from HCs. Only 10 circRNAs showed significantly upregulated in TB patients (fold change > 2; *p* < 0.01) (Table 2). The 10 upregulated circRNAs were selected for further analysis by qRT-PCR in the serum of 56 active TB patients, 20 community acquired pneumonia and 20 healthy controls during the Biomarker-selection phase. Compared with healthy controls and community acquired pneumonia, the expression level of circRNA_051239, circRNA_029965, and circRNA_404022 was significantly upregulated in TB patients (*P* < 0.01), while no significant difference in the other seven circRNAs was found (all *P* > 0.05, shown in Figure 3).

**TABLE 2:** circRNAs selected in Biomarker-screening phase by Arraystar Human CircRNA microarray V2 of pooled samples (three active TB patients, three healthy controls) with fold change = 2, and *p* < 0.01.

**CircRNA**	**Chrom**	**Strand**	**txStart**	**txEnd**	**CircRNA_type**	**Best_transcript**	**Gene symbol**	**FC (TB vs. HC)**	***p***
hsa_circRNA_051239	chr19	–	41938372	41945481	Exonic	uc010xwb.2	ATP5SL	5.358899153	0.007123
hsa_circRNA_029965	chr13	+	33306237	33344899	Exonic	NM_015032	PDS5B	5.174550937	0.004866
hsa_circRNA_404022	chr8	+	41466934	41469511	Exonic	NM_178819	AGPAT6	3.985615857	0.004119
hsa_circRNA_102116	chr17	–	47388673	47389404	Exonic	NM_014897	ZNF652	3.809132132	0.00888
hsa_circRNA_065793	chr3	+	50131152	50131308	Exonic	NM_005778	RBM5	3.175970107	0.004918
hsa_circRNA_104588	chr8	–	37727937	37735069	Exonic	NM_025151	RAB11FIP1	2.993253915	0.004175
hsa_circRNA_048148	chr19	+	1037623	1039064	Exonic	uc002lqu.3	CNN2	2.99009157	0.008405
hsa_circRNA_406174	chr22	–	28249524	28269803	Sense overlapping	NM_012399	PITPNB	2.934103983	0.002732
hsa_circRNA_100823	chr11	–	58317258	58322413	Exonic	NM_004811	LPXN	2.286347985	0.005376
hsa_circRNA_029301	chr12	–	124832370	124835283	Exonic	NM_006312	NCOR2	2.123749587	0.00054

### Biomarker-Validation Phase of the Serum CircRNAs for TB

**FIGURE 4:**
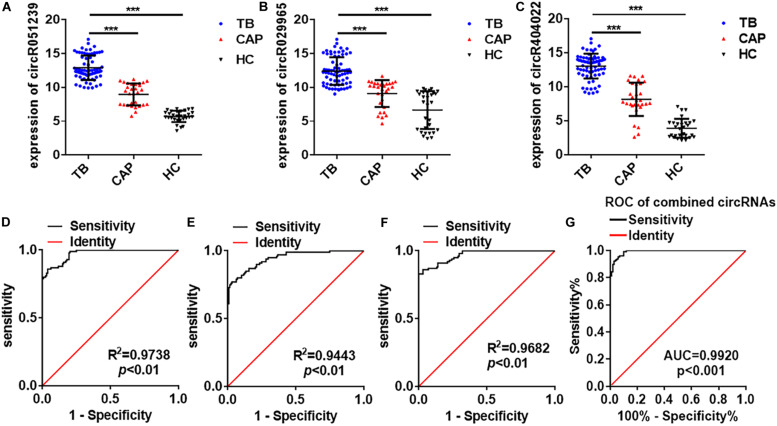
Serum circRNA expression signature for TB diagnosis in the validation phase. **(A–C)** The expression of circRNA_051239, circRNA_029965, and circRNA_404022 in serum from active TB patients (*N* = 72), CAP patients (*N* = 30), and healthy controls (*N* = 30) by qRT-PCR. **(D–F)** ROC curve analysis for circRNA_051239, circRNA_029965, and circRNA_404022 in serum from active TB patients (*N* = 128, including training set and validation set), CAP patients (*N* = 50, including training set and validation set), and healthy controls (*N* = 50, including training set and validation set). **(G)** ROC curve analysis for the combined circRNAs panel in serum from active TB patients (*N* = 128, including training set and validation set), CAP patients (*N* = 50, including training set and validation set), and healthy controls (*N* = 50, including training set and validation set). Each value is the mean ± SD; ***P < 0.001.

Next, these three circRNAs (circRNA_051239, circRNA_029965, and circRNA_404022) was further validated in a larger cohort, comprising 72 active TB patients, 30 community acquired pneumonia, 30 healthy controls by qRT-PCR (Figure 1). As the results in the Biomarker-selection phase, the concentrations of these three circRNAs (circRNA_051239, circRNA_029965, and circRNA_404022) in TB patients were significantly increased in the TB patients, compared to the community acquired pneumonia and healthy controls (Figures 4A–C).

### ROC Curve Analysis of Dysregulated CircRNAs

To assess the diagnostic value of these three serum circRNAs for TB detection, the ROC curve analysis was subsequently performed. As showed in Figures 4D–F, the area under the curve (AUC) values of these circRNAs were 0.9738 (95% CI, 0.9582–0.9893) for circRNA_051239, 0.9443 (95% CI, 0.9165–0.9721) for circRNA_029965, and 0.9682 (95% CI, 0.9496–0.9868) for circRNA_404022. Then, the diagnostic value of the combination of these three circRNAs was evaluated by a logistic regression model and found that the AUC was 0.9920 with *p* < 0.01 (Figure 4G). These results suggested circRNA_051239, circRNA_029965, and circRNA_404022 in the serum could serve as ideal potential biomarkers for TB diagnosis.

### CircRNA_051239 Was Significantly Upregulated in Drug-Resistant TB Patients

**FIGURE 5:**
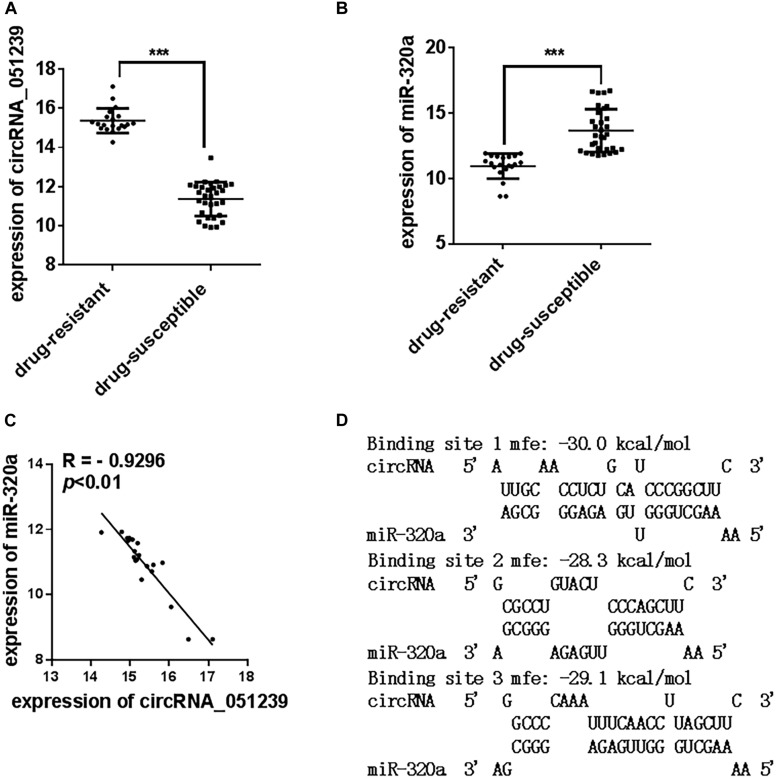
The expression level of circRNA_051239 and miR-320a in the serum of drug-resistant TB patients and drug-susceptible patients. **(A,B)** The expression level of circRNA_051239 and miR-320a in the serum of drug-resistant TB patients and drug-susceptible patients. **(C)** Spearman’s rank correlation scatter plot of circRNA_051239 and miR-320a in the serum of drug-resistant TB patients. Each value is the mean ± SD; ***P < 0.001. **(D)** Schematic descriptions of the hypothetical duplexes formed by miR-320a with circRNA_051239.

In the 128 TB patients, we found 20 patients were resistant to at least one drug, while 31 samples were pan-susceptible TB patients. We further analyzed the expression level of circRNA_051239, circRNA_029965, and circRNA_404022 in these drug-resistant TB patients and pan-susceptible TB patients. As the results showed in Figure 5A, circRNA_051239 was significantly increased in the drug-resistant group (Figure 5A), while the expression level of the other two circRNAs has no difference. More interestingly, Cui et al. (2017) reported levels of miR-320a were decreased in the drug-resistant group, compared to the pan-susceptible TB patients. We also evaluated the miR-320a level in our samples. As for the results reported by Cui et al. (2017), miR-320a significantly decreased in the drug-resistant group, compared to the pan-susceptible TB patients (Figure 5B). We conducted Spearmen correlation analysis to explore the correlation between levels of circRNA_051239 and miR-320a, the negative correlation was observed for circRNA_051239 and miR-320a (*R* = −0.9296, *p* = 0.0022) (Figure 5C).

We speculated circRNA_051239 might act as a ceRNA for miR-320a, and play a vital role in the TB drug-resistant progress. As predicted by RNAhydrid, miR-320a have three binding sites with circRNA_051239 (Figure 5D).

## Discussion

As newly discovered endogenous non-coding RNAs with covalently closed loop structures are resistant to RNase digestion (Memczak et al., 2013), circRNAs have been proved could serve as ideal potential biomarkers for tumors, Alzheimer’s disease, cardiovascular disease, and other diseases (Abu and Jamal, 2016). Recently, Fu et al. (2019) found circRNA_103017, circRNA_059914, and circRNA_101128 were increased in the peripheral blood mononuclear cells (PBMCs) from active tuberculosis (TB) patients, while circRNA_062400 was decreased in TB samples. Huang et al. (2018a) also identified the dysregulated circRNAs in the PBMCs from TB patients, and confirmed hsa_circRNA_001937, hsa_circRNA_009024, and hsa_ circRNA_005086 were significantly elevated and hsa_circRNA_102101, hsa_circRNA_104964, and hsa_circRNA_104296 were significantly reduced in PBMCs from TB patients as compared to healthy controls. Subsequently, they compared the expression level of circRNAs in the plasma from TB patients and healthy controls, the results showed the expression level of hsa_circ_0001204 and hsa_circ_0001747 were significantly decreased in the plasma of TB patients (Huang et al., 2018b), and hsa_circ_0001953 and hsa_circ_0009024 were remarkably increased in the plasma of TB patients (Huang et al., 2018c). Yi et al. (2018) reported hsa_circRNA_103571 exhibited significant decrease in the plasma of active TB patients and showed potential interaction with active TB-related miRNAs, such as miR-29a and miR-16. As for the RNA isolation, transportation and preservation of PBMCs and plasma is too complexity to do quality control, the circRNAs in the PBMC and plasma could not serve as ideal biomarkers for TB diagnosis in clinical application. Compare to PBMCs and plasma, serum is an ideal material for molecular diagnosis. In order to investigate the potential diagnosis value of circRNAs in the serum for TB diagnosis, we characterized the profile of differentially expressed circulating circRNAs in the serum of active TB patients, CAP patients and healthy controls by circRNA microarray and qRT-PCR. As the results in the PBMCs and plasma, the expression profile of circulating circRNAs in the serum of active TB patients showed significantly different from the healthy controls, and we found that circRNA_051239, circRNA_029965, and circRNA_404022 significantly increased in the serum of TB patients and could serve as ideal biomarkers for TB diagnosis with the AUC of all of the three circRNAs is larger than 0.9.

Drug-resistant TB is one of the biggest threats for global TB control and remains a major public health concern in many developing countries. In 2018, more than 500,000 new cases of multidrug-resistant TB and an additional 100,000 cases with rifampicin-resistant TB were identified (Reinhart et al., 2012). Until now, there is still no biomarker to identify whether the TB patients was drug-resistant. In 2017, Cui et al. (2017) reported miR-320a in the plasma significantly decreased in the drug-resistant TB patients. We also evaluated the expression level of miR-320a in the serum and found miR-320a in the serum also significantly decreased in the drug-resistant TB patients. However, the expression level of miR-320a in the serum/plasma altered in kinds of diseases. Such as, plasma miR-320a showed significantly decreased in the Arrhythmogenic CardioMyopathy (ACM), compared to the healthy controls (Sommariva et al., 2017). The concentrations of plasma miR-320a were decreased in patients with colorectal cancer and benign lesions (polyps and adenoma) compared with healthy controls, but increased in inflammatory bowel disease (IBD) (Fang et al., 2015). In the present study, circRNA_051239 was significantly upregulated in serum derived from drug-resistant TB patients compared to drug-susceptible patients, implying circRNA_051239 as a potential marker for discriminating drug-resistant TB patients. Moreover, we found the expression levels of circRNA_051239 and miR-320a showed negative correlation (Figure 5C). Recent studies have confirmed circRNAs play essential roles in various physiological and pathological processes via acting as a ceRNA for miRNAs to relive the repression of miRNAs for their target genes (Thomson and Dinger, 2016). Here, circRNA_051239 was speculated to sponge miR-320a to release the target genes of miR-320a, and play a vital role in the TB drug-resistant progress. However, it needs to be further confirmed.

In summary, our study firstly provided a profile of circulating circRNAs in the serum of TB patients and healthy controls. We found circRNA_051239, circRNA_029965, and circRNA_404022 significantly increased in the serum of TB patients and could serve as ideal biomarkers for TB diagnosis. Moreover, circRNA_051239 could distinguish drug-resistant TB patients from pan-susceptible TB patients.

## Data Availability Statement

All datasets generated for this study are included in the article/Supplementary Material. Raw and processed data are stored in the laboratory and are available upon request.

## Ethics Statement

The studies involving human participants were reviewed and approved by Medical Ethics Committee, Nanjing Drum Tower Hospital, The Affiliated Hospital of Nanjing University Medical School. The patients/participants provided their written informed consent to participate in this study.

## Author Contributions

FH and JW designed the experiments. HL, GL, WW, XJ, SG, and XY performed the experiments and analyzed the results. JW helped to analyze the microarray of circRNA and made critical reading of the manuscript. XY wrote the manuscript.

## Conflict of Interest

The authors declare that the research was conducted in the absence of any commercial or financial relationships that could be construed as a potential conflict of interest.

## Supplementary Material

TABLE S1 The circRNA expression profile in the serum of TB patients and healthy controls by circRNA Microarray.
